# Machine learning increases the prediction of stroke for Chinese hypertensive patients

**DOI:** 10.3389/fmicb.2026.1737655

**Published:** 2026-01-23

**Authors:** Ying Zhou, Wanshu Deng, Wentao Wang

**Affiliations:** 1 Department of Cardiology, The First Hospital of China Medical University, Shenyang, China; 2 Department of Cardiology, The First Affiliated Hospital of Dalian Medical University, Dalian, China; 3 Department of Gastric Surgery, Liaoning Cancer Hospital and Institute (Affiliated Cancer Hospital of Dalian University of Technology, Cancer Hospital of China Medical University), Shenyang, China

**Keywords:** hypertension, ML method, prediction, stroke, XGboost

## Abstract

**Background:**

We aim to construct a machine learning (ML) model to predict stroke risk in patients with hypertension.

**Methods:**

In all, 68 variables, including demographic information, medical history and medication use, lifestyle, anthropometry laboratory tests, electrocardiography, and echocardiography, were selected for baseline analysis. Of these, 10 optimal variables were selected by Recursive feature elimination (RFE) and then the model was trained and tested using eXtreme Gradient Boosting (XGBoost). A 10- fold cycle of cross-validation was used during the process. Next, XGBoost was used to develop a prediction model. Four traditional Cox regression models including the China-PAR Score and the Framingham Stroke Risk Score model were established and compared with the ML model. Finally, the results of the performance assessment of the models were compared using C-statistics for discrimination and Brier score for calibration.

**Results:**

In all, we included 5,197 hypertensive participants (mean age = 57.16 ± 10.20 years) from the Northeast China Rural Cardiovascular Health Study (NCRCHS). Of these, end point events occurred in 294 patients (5.7%, 185 males and 109 females) during a mean follow-up period of 4.26 ± 1.03 years. Using RFE, 10 variables were selected to construct the XGBoost model. The ML model demonstrated better discrimination than the best performing Cox regression model [C-statistic 0.967 (95% CI, 0.956, 0.978) vs. 0.781 (95% CI, 0.772, 0.785), respectively] with an acceptable calibration (Brier score = 0.053).

**Conclusion:**

Using the ML method, we constructed a high-precision prognostic model to predict stroke risk in patients with hypertension. This model exhibited a better classification effect and better performance compared with the traditional risk scales. The model could be used in clinical practice to achieve early prevention and intervention of stroke.

## 1 Introduction

Globally, stroke is among the most serious public health problems since it is the main cause of disability and the second leading cause of death (Mathers and Loncar, 2006). In 2017, a survey reported >13 million stroke cases in China (Wang et al., 2017). According to the National Disease Surveillance Points System, 1.6 million people die due to stroke in China every year, which is almost one-third of the total deaths from stroke worldwide (Liu et al., 2011; Feigin et al., 2015; Zhou et al., 2016; Wang et al., 2017). The incidence and total mortality of stroke in China are rising every year (Wang et al., 2020).

China has the largest number of hypertensive patients. Among Chinese adults ≥18 years old, approximately 244.5 million individuals have hypertension (HTN) and 435.3 million have pre-HTN. Strikingly, HTN is an important risk factor for stroke, especially in China. Over 60% of the patients with acute stroke present with high blood pressure (Miller et al., 2014). In Chinese hypertensive patients, every 10 mmHg increase in systolic blood pressure is associated with 1.44-fold and 1.5-fold risk for ischemic and hemorrhagic stroke, respectively. Moreover, the stroke risk is remarkably higher among Chinese hypertensive patients than their Caucasian counterparts (risk ratio is 2.58) (Zhang et al., 2006). Therefore, the burden of stroke is huge in China. Thus, accurate prediction of stroke in the hypertensive population is critical for stroke prevention in China.

To date, some prediction models of stroke, such as the Framingham Stroke Risk Score (FHS), (Kannel et al., 1976; D'Agostino et al., 1994, 2008; Zhou et al., 2017) the European Pooled Cohort Equations (PCE) (Goff et al., 2014). and the Q Stroke score in the United Kingdom, have been developed (Hippisley-Cox et al., 2013). However, the applicability of these models in the Chinese population has always been questioned with overestimated risks (Liu et al., 2004; Chia et al., 2014; Jiang et al., 2020; Li J. et al., 2021). Although the Chinese Multi-provincial Cohort Study (CMCS) (Liu et al., 2004; Wang et al., 2016) and the Prediction for ASCVD Risk in China (China-PAR model) were based on the Chinese population, (Yang et al., 2016; Xing et al., 2019) their accuracy and application were compromised by the limited risk factors considered by them and the traditional analysis method used.

Machine learning (ML) algorithms have shown good performance in the diagnosis, (Chen, 2022) treatment (Chi et al., 2022), and prognosis (Motwani et al., 2017; Segar et al., 2019; Angraal et al., 2020; Wu et al., 2020; Shu et al., 2021) of cardiovascular diseases (CVDs). Nonetheless, no stroke prediction model to date has been developed from the general Chinese hypertensive cohort using ML algorithms (Wu et al., 2020; Yang et al., 2021). Hence, this study aims to develop a ML method-based model for more accurate prediction of stroke risk in Chinese hypertensive patients compared with traditional models.

## 2 Methods

### 2.1 Patient cohort

The Northeast China Rural Cardiovascular Health Study (NCRCHS) is a prospective cohort study conducted in the rural areas of Northeast China, whose inclusion criteria and design are described in the previous article (Guo et al., 2021a). In all, 11,956 participants aged ≥35 years were recruited from Dawa, Zhangwu, and Liaoyang counties of Liaoning province between 2012–2013 using a multi-stage, randomly stratified cluster-sampling scheme. The participants were followed up in 2015 and 2017. The NCRCHS was approved by the Ethics Committee of China Medical University (Shenyang, China). All participants provided written informed consent.

In this study, we included data from 5,260 participants of the NCRCHS study. Of these, 63 patients with missing or abnormal values were excluded. As a result, 5,197 hypertensive patients were included in the final analyses (Supplementary Figure S1).

### 2.2 Study variables and data collection

The data collection procedure is described in detail in our previous paper (Li Z. et al., 2021). The patients' demographic characteristics, medical history and medication use, lifestyle factors, and other information were obtained at baseline through interviews by trained research staff using a standardized validated questionnaire. Indices such as weight, height, and waist circumference (WC). Body mass index (BMI) was computed as body weight in kilograms divided by the square of the height in meters. Blood pressure (BP) was assessed thrice after a 5-min rest using an automatic electronic sphygmomanometer (HEM-907; Omron, Kyoto, Japan) and averaged. HTN was defined according to the JNC 7 report as systolic blood pressure (SBP) ≥140 mmHg, diastolic blood pressure (DBP) ≥90 mmHg, and/or the use of antihypertensive medications (Chobanian et al., 2003). We collected blood samples after at least 12 H of fasting to determine the plasma levels of fasting glucose (FPG), triglycerides (TG), high-density lipoprotein cholesterol (HDL-C), uric acid, estimated glomerular filtration rate (eGFR), and blood routine biochemical indicators. Standard 12-lead electrocardiograms (ECGs) were used with a MAC 5500 (GE Healthcare, Little Chalfont, UK) as previously described (Li Z. et al., 2021). The ECGs were analyzed automatically, including QRS duration, PR duration, P axis, R axis, T axis, left ventricular hypertrophy (LVH) ECG (define per Sokolow–Lyon criteria), and QT interval (Framingham). Atrial fibrillation (AF) was defined as having a previous history of AF or an ECG suggestive of AF. Echocardiography was performed for all participants based on the American Society of Echocardiography guidelines, which were consistent with our previous study (Li T. et al., 2021). A Doppler echocardiography (Vivid; GE Healthcare, Connecticut, USA) with a 3.0-MHz transducer (Vivid, GE Healthcare, USA), including M-mode, two-dimensional, spectral, and color Doppler was used. Aortic dimension (AD), left atrial diameter (LAD), left ventricular end-diastolic internal dimension (LVIDd), left ventricular end—systolic internal dimension (LVIDs), interventricular septal thickness (IVSd), posterior wall thickness (PWTd), left ventricular ejection fraction (LVEF), E wave, and A wave were measured. In all, we selected 68 variables for model construction (Table 1), including subsets of characteristics related to the course, prognosis, hypertension-related target organ damage, and complications.

**Table 1:** Variables used for machine learning.

**Category**	**Variables**
Demographics	Age, gender, ethnicity, marriage, education, family income
Medical history and medication use	CHD, heart failure; AF, hemorraghic stroke; ischemic stroke; PCI or CABG, DM, renal dysfunction, all antihypertensive medication, ACEI, ARB, beta.blockers, CCB, diuretics, statins, asprin, warfarin, family history of CHD, family history of stroke
Lifestyles	Smoking, drinking, regular exercise, sleeping duration, salt intake
Anthropometry	BMI, WC, TW, SBP, DBP, PP, HR
Lab tests	HDL C, LDL C, TC, TG, FPG, K, Na, Mg, Ca, eGFR, Uric acid, WBC, Hb, PLT, ALT
Electrocardiographic (ECG)	PR duration; QRS duration; QT duration; P axis, QRS axis, T axis; LVH ECG; AF ECG
Echocardiography	AD, LAD, LVIDd, LVIDs, IVSd, PWTd, LVEF, E wave, A wave

CHD, angina or myoinfarction; ACEI, DM, Diabetes Mellitus; angiotensin converting enzyme; ARB,angiotensin-II receptor blockers; CCB, calcium channel blockers; BMI, body mass index; WC, waist circumference; TW, hip circumference; SBP, systolic blood pressure; DBP, diastolic blood pressure; PP, pulse pressure; HR, heart rate; HDL C, high density lipoprotein cholesterol; LDL C, low density lipoprotein cholesterol; TC, total cholesterol; TG, triglycerides; FPG, fasting glucose; K, potassium; Ca, sodium; eGFR, estimated glomerular filtration rate; LVH ECG, left ventricular hypertrophy electrocardiographic; AF ECG, atrial fibrillation electrocardiographic; AD, aortic dimension; LAD, left atrial diameter; LVIDd, left ventricular end—diastolic internal dimension; LVIDs, left ventricular end-systolic internal dimension; IVSd, interventricular septal thickness; PWTd, posterior wall thickness; LVEF, left ventricular ejection fraction.

### 2.3 Follow-up

During follow-up, we collected the end point events of new fatal or non-fatal strokes. According to the World Health Organization (WHO) Multinational Monitoring of Trends and Determinants in CVD criteria, stroke was defined as rapidly developing signs of focal or global disturbance of cerebral functions lasting for >24 H (unless interrupted by surgery or death) with no apparent non-vascular cause (Asplund et al., 1988). Chronic cerebral vascular disease and transient ischemic attack were excluded. We collected medical records and death certificates for all participants who were possibly diagnosed or died. All information was independently reviewed and judged by the end point assessment committee.

### 2.4 Cox regression model construction

We established four Cox regression models and compared them with the ML model. For one Cox proportional hazards model, Cox proportional hazards analysis was performed on all variables, and redundant variables were eliminated via the forward conditional stepwise selection method in Cox regression. Two regression models selected variables established in the China-PAR Score (Yang et al., 2016) and the Framingham Stroke Risk Score (D'Agostino et al., 1994). The last Cox model used LASSO regression to filter the variables.

### 2.5 Ml model construction and calculation

To avoid data leakage, all data preprocessing and model construction procedures were conducted strictly within each training subset of a 10-fold cross-validation framework, while the corresponding validation subset was used only for model evaluation. Missing data were handled using multivariate imputation by chained equations (MICE), and outlier detection procedures were performed on the training data in each fold, with the derived parameters applied to the validation data.

The machine learning workflow, as shown in Supplementary Figure S1, involved feature selection using RFE, model training, and testing with XGBoost within a 10-fold cross-validation cycle. RFE was performed independently in each training subset, and validation errors across all folds were calculated to identify the optimal feature combination with the lowest average error. After feature selection, Synthetic Minority Over-sampling Technique (SMOTE) was applied only to the training data to balance class distribution, whereas validation data retained their original distribution, and SMOTE was implemented using the k-nearest neighbors approach (*k* = 5). Subsequently, XGBoost was used to develop the prediction model, a gradient tree boosting-based classifier that aggregates multiple weak learners into a strong learner, and was trained using the *gbtree* booster (max_depth = 4, learning rate = 0.05, n_estimators = 300, subsample = 0.8, and colsample_bytree = 0.8). All machine learning analyses were implemented in the open-source R software (version 4.1.1).

### 2.6 Statistical analysis and performance measures

Missing laboratory values were imputed using the mice package in R (*m* = 5 imputations, max it = 10 iterations), with the imputation model fitted on the training data in each fold and then applied to the corresponding validation data. For outliers, the local outlier factor (LOF) was used for numerical variables and the attribute value frequency (AVF) algorithm for categorical variables. Continuous variables were represented as mean ± standard deviation (SD) and compared using the *t*-test or Mann–Whitney *U* test. Categorical variables were represented as frequency (*n*) and proportion (%) and compared using the chi-square test. C-statistics was used to evaluate the performance of the models (DeLong et al., 1988). Calibration of the models was evaluated by the Brier score method (range, 0– 1) (Brier, 1950) and the numbers of observed and predicted events proportion were grouped according to the decile of predicted risk (Liu et al., 2004). A *P*-value < 0.05 was considered statistically significant. Decision curve analysis (DCA) was performed to assess the clinical net benefit of each model at different threshold probabilities, providing a visual comparison of their potential clinical utility.

SHapley Additive exPlanations (SHAP) is a framework based on the additive feature attribution method that explains the output of the XGBoost model. A positive SHAP value indicates that the feature has a positive effect, while a negative SHAP value indicates that the feature reduces the outcome value and has a negative effect. This method can output the importance ranking of the features as well as the relationship between the features and the outcomes. SHAP-force plot was used to visualize the impact of individual feature values on the model's prediction for each observation. Descriptive analyses and comparisons between clinically defined groups were performed using R 4.1.1.

## 3 Results

### 3.1 Participants' characteristics data description

In all, 5,197 individuals with hypertension were included in the study. Of these, 49.4% were males, 50.6% were females, and the mean age was 57.16 years. End point events occurred in 294 (5.7%) patients during a mean follow-up period of 4.26 ± 1.03 years, among which 185 were males and 109 were females. Table 2 shows the distribution of the risk factors. Individuals with end points were older and had higher WC, SBP, DBP, FBG, QTc Framingham, AD, LAD, IVSd, LVIDs, PWTd, and A wave compared with those without end points. Furthermore, they take antihypertensive medications more frequently and were more likely to have a stroke history and left ventricular hypertrophy (LVH) ECG. In contrast, eGFR, R axis, LVEF, and E wave were lower in individuals with end points than those without end points.

**Table 2:** Baseline characteristics.

**Characteristics**	**Total (*n* = 5,197)**	**Patients without end points (*n* = 4,903)**	**Patients with end points (*n* = 294)**	***P* value**
Male	2,566 (49.4%)	2,381 (48.6%)	185 (62.9%)	< 0.001
Age (years)	57.16 ± 10.20	56.84 ± 10.20	62.50 ± 8.66	< 0.001
CHD history	345 (6.64%)	316 (6.45%)	29 (9.86%)	0.022
AF history	62 (1.19%)	56 (1.14%)	6 (2.04%)	0.27
Hemorrhagic stroke history	62 (1.19%)	57 (1.16%)	5 (1.70%)	0.583
Ischemic stroke history	258 (4.96%)	225 (4.59%)	33 (11.2%)	< 0.001
DM history	322 (6.20%)	300 (6.12%)	22 (7.48%)	0.346
PCI/CABG	9 (0.17%)	8 (0.16%)	1 (0.34%)	1
Antihypertentisve medication with in 2 weeks	1,510 (29.1%)	1,370 (27.9%)	140 (47.6%)	< 0.001
Family stroke (Yes)	1,002 (19.3%)	927 (18.9%)	75 (25.5%)	0.005
Current smoke (Yes)	1,834 (35.3%)	1,716 (35.0%)	118 (40.1%)	0.073
Exercise (Yes)	1,270 (24.4%)	1,201 (24.5%)	69 (23.5%)	0.691
Sleep duration (h/d)	7.26 ± 1.74	7.27 ± 1.72	7.07 ± 1.93	0.155
Salt (g/d)	7.07 ± 5.12	7.22 ± 8.43	7.02 ± 4.15	0.406
BMI (kg/m^2^)	25.58 ± 3.61	25.57 ± 3.61	25.64 ± 3.56	0.779
Mean WC (cm)	84.75 ± 9.65	84.66 ± 9.59	86.20 ± 10.55	0.009
Mean SBP (mmHg)	159.31 ± 19.38	158.69 ± 18.97	169.81 ± 22.79	< 0.001
Mean DBP (mmHg)	88.95 ± 11.12	88.75 ± 10.94	92.32 ± 13.34	< 0.001
PP (mmHg)	70.35 ± 17.43	69.93 ± 17.28	77.34 ± 18.43	< 0.001
Mean pulse (bpm)	79.42 ± 14.10	79.45 ± 14.09	78.89 ± 14.22	0.511
TC (mmol/L)	5.43 ± 1.11	5.42 ± 1.11	5.50 ± 1.12	0.268
HDL C (mmol/L)	1.43 ± 0.41	1.44 ± 0.41	1.41 ± 0.43	0.076
LDL C (mmol/L)	3.11 ± 0.86	3.10 ± 0.86	3.18 ± 0.88	0.086
FPG (mmol/L)	6.14 ± 1.87	6.13 ± 1.86	6.39 ± 2.07	0.007
PLT (10^9^/L)	215.54 ± 59.87	216.07 ± 59.99	206.67 ± 57.14	0.009
Ca (mmol/L)	2.34 ± 0.14	2.34 ± 0.14	2.33 ± 0.15	0.754
eGFR (mL/min/1.73 m^2^)	91.14 ± 15.08	91.50 ± 15.02	85.19 ± 14.97	< 0.001
QTc Framingham (ms)	422.08 ± 22.04	421.76 ± 22.02	427.40 ± 21.57	< 0.001
LVH ECG (Yes)	930 (17.9%)	856 (17.5%)	74 (25.2%)	0.001
AF ECG (Yes)	30 (0.58%)	27 (0.55%)	3 (1.02%)	0.525
LAD (cm)	3.36 ± 0.72	3.35 ± 0.72	3.53 ± 0.61	< 0.001
IVSd (cm)	0.94 ± 0.43	0.94 ± 0.42	1.01 ± 0.52	< 0.001
PWTd (cm)	0.90 ± 0.35	0.90 ± 0.33	0.98 ± 0.55	< 0.001

Data are expressed as the mean ± SD or as *n* (%).

### 3.2 Model evaluation and comparison

**Figure 1:**
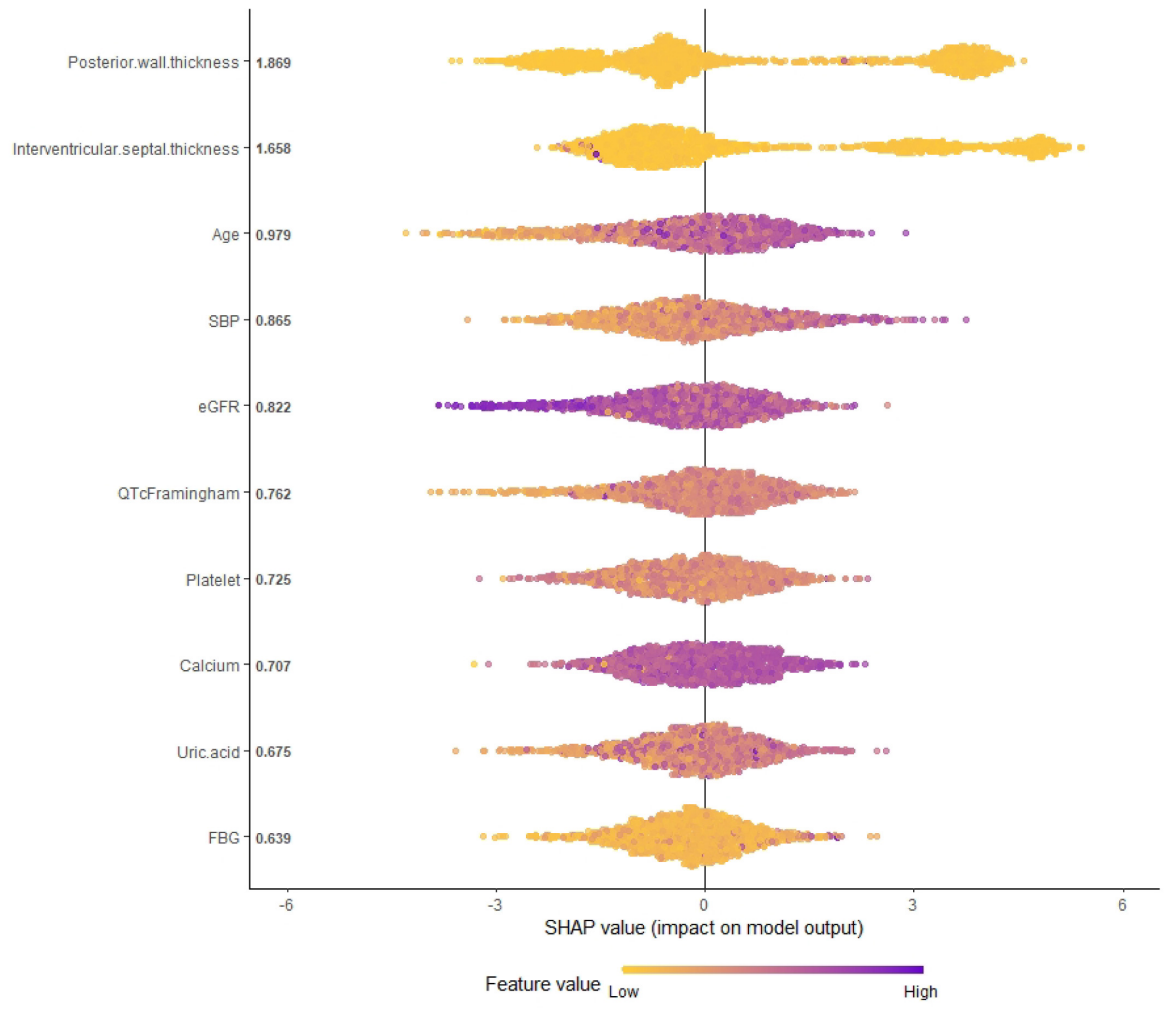
SHAP summary plot of the XGBoost model.

Finally, we selected and analyzed 10 variables for the ML model by the RFE and XGBoost combination. The 10-variable combination included PWTd, IVSd, age, SBP, eGFR, QTc Framingham, platelet, calcium, uric acid, and FBG. We constructed a SHAP summary plot of the XGBoost model (Figure 1) to identify the importance of each feature in the prediction model. We identified that PWTd, IVSd, age, and SBP were the most important risk factors for stroke (Figure 1). In contrast, eGFR was associated with a decreased risk of stroke. We further generated a SHAP force-style plot for a representative patient to illustrate the individualized prediction of the XGBoost model, showing how each feature contributed to the predicted stroke recurrence risk (Supplementary Figure S2).

**Figure 2:**
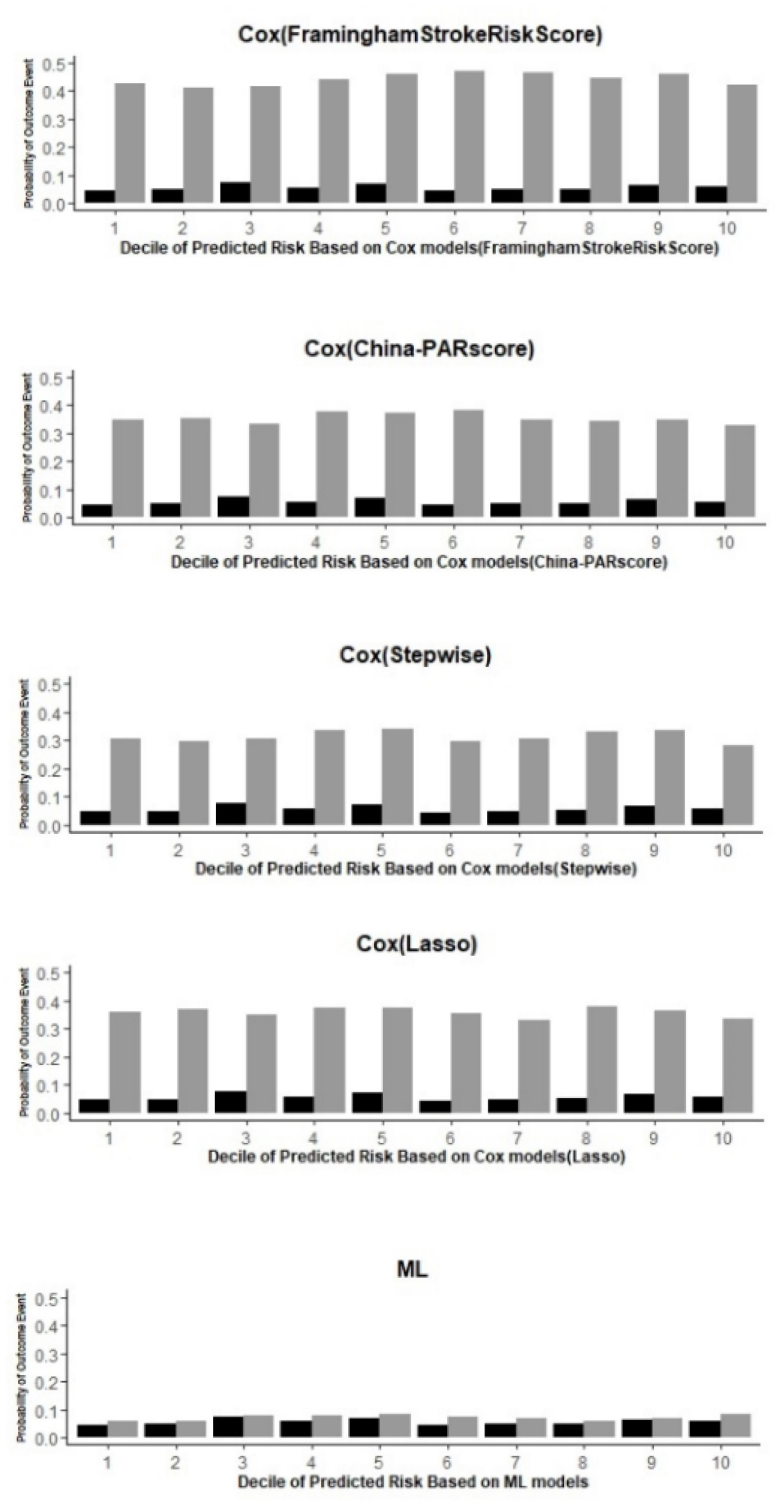
Prediction of outcome events and observed end points in each decile using the ML and 4 Cox models.

The results of the performance assessment were compared using C-statistics for discrimination and Brier score for calibration (Table 3) with 10-fold cycle of cross-validation. For predicting the end point events, the C-statistics was highest for the ML model [0.967 (95% CI, 0.956, 0.978)] among the five models. The four Cox regression models were similar to each other and their C-statistics were: Cox Regression (Framingham Stroke Risk Score) [0.747 (95% CI, 0.734, 0.762)], Cox Regression (China-PAR score) [0.725 (95% CI, 0.707, 0.731)], Cox Regression (Stepwise) [0.781 (95% CI, 0.772, 0.785)], and Cox Regression (LASSO) [0.764 (95% CI, 0.757, 0.772)]. Therefore, our ML model based on XGBoost had a better classification effect and better performance compared with the traditional risk scales.

**Table 3:** Comparison of model variables and performance values.

**Model**	**Cox regression (Framingham stroke risk score)**	**Cox regression (China-PAR score)**	**Cox regression (stepwise)**	**Cox regression (lasso)**	**Ml**
Variable	Age, sex, antihypertentisvemedication, SBP, Current smoke, DM history, LVH ECG, AF, prior CVD	Age,SBP, current smoke, DM history, TC, HDL C,WC, family stroke	District,sex,age, Antihypertentisve medication, Physical activity, Sleep duration, Salt intake, SBP,PP, Uric acid, potassium, eGFR, QTcFramingham, Left atrial diameter, IVSd	District, sex, age, antihypertentisvemedication, SBP, DBP, eGFRQTcFramingham, left atrial diameter	PWTd, Age, IVSd, SBP, eGFR, FBG, Calcium, QTcframingham, Uric acid, platelet
C	0.737	0.725	0.781	0.764	0.967
95%CIforC	(0.727, 0.748)	(0.707, 0.731)	(0.772, 0.785)	(0.757, 0.772)	(0.956, 0.978)
BS	0.055	0.055	0.056	0.056	0.053
95%CiforBS	(0.048, 0.062)	(0.049, 0.063)	(0.049,0.062)	(0.048,0.062)	(0.038, 0.068)

The Brier score for the ML model was 0.053, indicating a good calibration between the estimated predicted risk and the observed 4.26 years risk. Calibration was also assessed by comparing the predicted and observed risks in each decile (Table 3). The largest difference for ML was small (2.9% in the 6th decile) compared with that of Cox (Framingham Stroke Risk Score) (42.7% in the 6th decile), Cox (China-PAR score) (33.8% in the 6th decile), Cox (stepwise) (27.9% in the 8th decile), and Cox (LASSO) (32.9% in the 8th decile).

**Figure 3:**
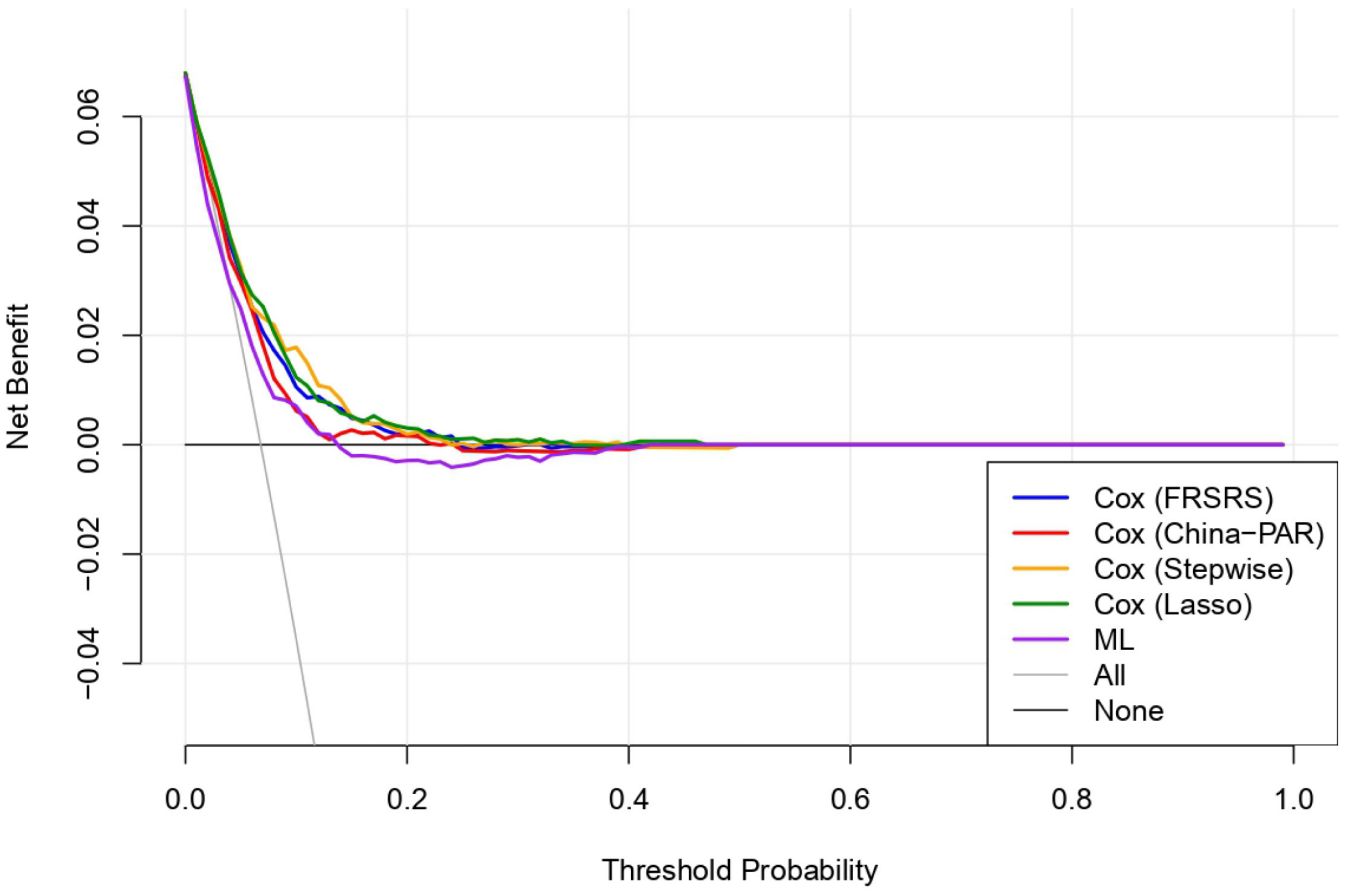
Decision curve analysis (DCA) comparing the clinical net benefit of five models.

Decision curve analysis (DCA) showed that all five models provided net clinical benefit mainly at low threshold probabilities (approximately 0–0.15), whereas net benefit rapidly approached zero as the threshold increased (0.15–0.20), indicating limited utility at moderate-to-high thresholds (Figure 3). The DCA curves largely overlapped across the evaluable range, suggesting that the differences in net benefit among models were small and not clinically apparent; importantly, the ML model demonstrated net benefit comparable to the Cox regression models with only marginal variations across thresholds.

## 4 Discussion

This study presents a novel ML technique that integrates demographic characteristics, basic information, blood biochemical indicators, electrocardiographic variables, and echocardiographic indicators to efficiently predict the risk of stroke among Chinese hypertensive patients. We found that the performance of the ML model was better than that of the four Cox regression models with a significantly high C-statistic.

ML methods are powerful tools and are increasingly applied for diverse medical applications to predict disease outcomes. XGBoost, an advanced method, has been consistently shown to be one of the best ML methods in supervised learning tasks. This algorithm can capture complex and non-linear interactions between variables. Additionally, it can learn its splitting direction for samples with missing values automatically and reduce overfitting and calculation.

Nonetheless, only a few ML prognostic models have been reported for hypertensive patients. Additionally, these models have certain limitations. For instance, Wu et al. used the ML method to construct a prognostic model for predicting the risk of hypertension in young patients. This ML approach was comparable with Cox regression and was outperformed the recalibrated FRS model (Wu et al., 2020). Nonetheless, the study focused on the young population and only 508 samples were enrolled among which 42 had end point events; hence, generalizability to all age individuals remains to be studied. Additionally, Yujie Y et al. constructed a stroke risk prediction model for patients with hypertension based on large-scale electronic medical record systems (EMRs) and proved that the ML models perform better than the traditional methods (Yang et al., 2021). Nevertheless, since it was a retrospective study based on EMRs, numerous values were missing; hence, some important traditional scales were not included in this model. Fortunately, this was a prospective study based on a large-scale cohort and the collection of basic information was reliable and the measurement results were unified. Thus, the results apply to a wide range of populations, especially Northeast China that has a high stroke incidence.

In this research, we selected 10 variables for constructing the ML model. Of these, eight variables (PWTd, IVSd, eGFR, FBG, calcium, QTc Framingham, uric acid, and platelet) were different from the traditional Cox models and were seldom discussed for the risk prediction of stroke. According to the SHAP summary plot, PWTd and IVSd had the highest predictive value for stroke, while the predictive value of FBG was much lower. PWTd and IVSd are indices of LVH. LVH in echocardiography is an independent predictor of incident CVDs (Gupta et al., 2010; Leigh et al., 2016). In addition, it was proved that hypertension with LVH was an extremely high-risk factor for CVDs. Li Z. et al. (2021) found that ventricular septal thickness in echocardiography should be considered when constructing risk prediction models for CVDs. In addition, studies revealed that eGFR is independently associated with cardiovascular events, although it may not be recognized as a major risk factor as SBP (Go et al., 2004; Chung et al., 2007; Sosner et al., 2015). This could be attributed to atherosclerosis, which can influence the renal blood vessels leading to renal insufficiency. Guo et al. (2021b) found that each 10 ms increase in the QTc interval was associated with an HR of 1.12 for stroke. Uric acid ranked ninth on the list of influencing factors. Several studies have shown that uric acid was an independent risk factor for ischemic stroke, especially for predicting ischemic stroke in Chinese hypertensive patients (Zhang et al., 2020; Dong et al., 2021). However, some studies failed to identify significant evidence between uric acid levels and the risk of the first stroke in Chinese adults with hypertension (Shi et al., 2017; Hu et al., 2021); hence, further studies are needed to validate the relationship. An abnormal T axis was identified to be an independent risk factor for CVD; hence, ECG monitoring to identify T-wave axis deviation can be an early indicator of CVD and help avoid cardiac events.

Inevitably, the study has several limitations. (1) We constructed a ML model based on XGBoost to compare with the traditional Cox regression models since it was previously proven to be better than other ML models (Yang et al., 2021). However, other non-linear ensemble methods, such as SVM, decision trees, and KNN classifiers, which also outperformed the traditional models were not included. (2) Potential predictors (Naganuma et al., 2013; Li et al., 2015; Su et al., 2020) of stroke, such as cranial imaging, were not collected either at baseline or follow-up. Meanwhile, competing risks may lead to overestimation of stroke risk, and we will subsequently apply the Fine-Gray model for sensitivity analysis. (3) Similar to previous articles, this research was performed on a rural population of northeast China without validation in independent cohorts. However, we used 10-fold cycle of cross-validation to compensate for the lack of external verification. It has been confirmed and used in previous research (Motwani et al., 2017; Juhola et al., 2021). 10-fold cycle of cross-validation can reduce the variance in prediction error and minimize overfitting and optimism bias. In addition, the follow-up time of 4.26 ± 1.03 years was short and a long-term follow-up is required.

## 5 Conclusion

In summary, we used an ML method to construct a prognostic model with 10 selected variables for predicting the risk of stroke in patients with hypertension. The XGBoost model had better performance compared with the traditional models. The ML predictive model may be useful to identify hypertensive patients developing stroke so that targeted prevention strategies can be carried out and it is highly expected to be applied in clinical practice.

## Data Availability

The raw data supporting the conclusions of this article will be made available by the authors, without undue reservation.

## Supplementary material

Supplementary Figure S1 The process for building and evaluating the performance of a ML method.

Supplementary Figure S2 Individual-level explanation of the XGBoost model prediction using a SHAP force-style plot. the model output is shown on the log-odds scale, starting from the base value (dashed line) and progressing to the final predicted risk of 18.1%. Each bar represents the contribution of an individual feature to the patient-specific prediction, with colors indicating whether the feature increases or decreases the predicted risk.
