# Supplementary figures and images for: Machine learning increases the prediction of stroke for Chinese hypertensive patients
Source: Front Microbiol. 2026 Jan 23;17:1737655. doi: 10.3389/fmicb.2026.1737655 (PMC12880818; doi:10.3389/fmicb.2026.1737655)

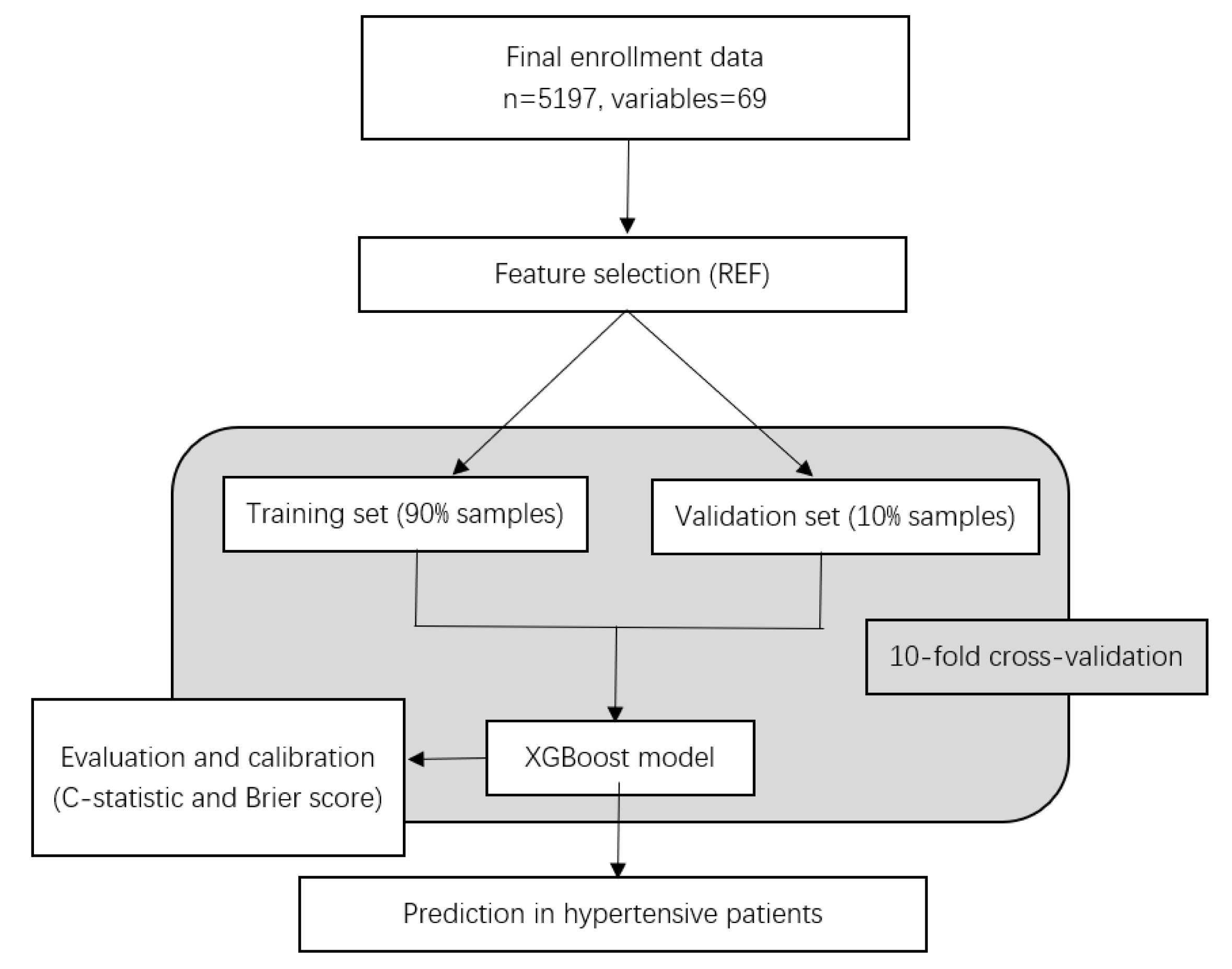

Supplement: Supplementary Figure S1 — The process for building and evaluating the performance of a ML method. [file Image_1.tif]

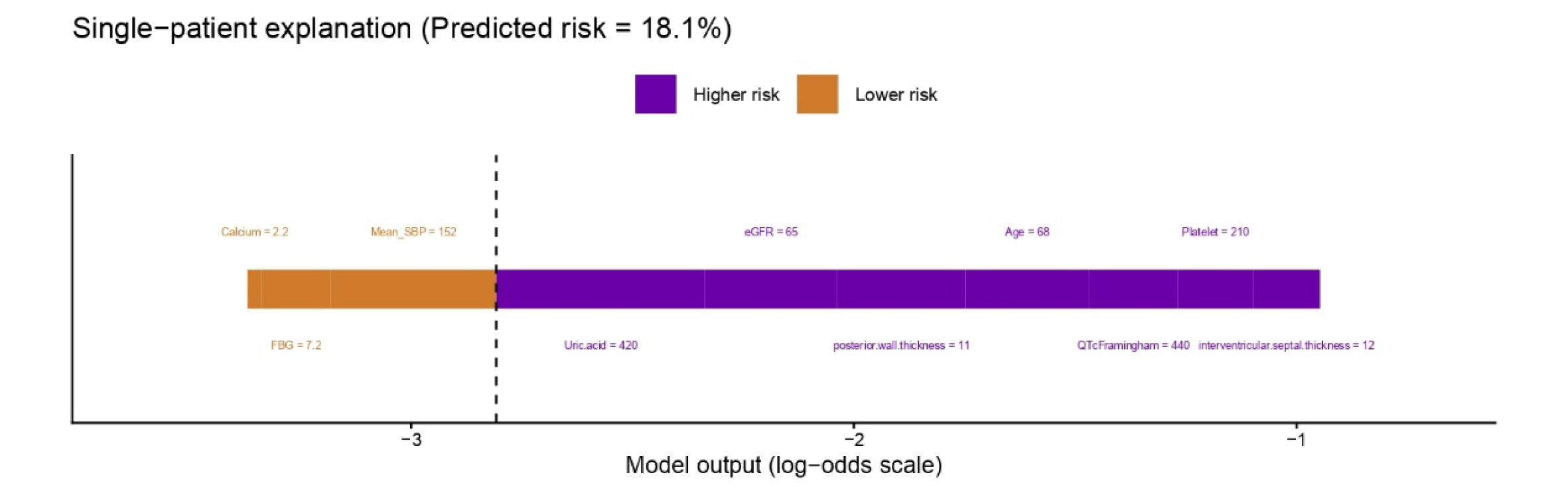

Supplement: Supplementary Figure S2 — Individual-level explanation of the XGBoost model prediction using a SHAP force-style plot. the model output is shown on the log-odds scale, starting from the base value (dashed line) and progressing to the final predicted risk of 18.1%. Each bar represents the contribution of an individual feature to the patient-specific prediction, with colors indicating whether the feature increases or decreases the predicted risk. [file Image_2.tif]
